# A Pulsed‐Dose Study Evaluating Chronic Toxicity of Chlorothalonil to Fish: A Case Study for Environmental Risk Assessment

**DOI:** 10.1002/etc.4421

**Published:** 2019-06-19

**Authors:** Mick Hamer, Samuel K. Maynard, Suzanne Schneider

**Affiliations:** ^1^ Syngenta Jealotts Hill International Research Station Bracknell Berkshire United Kingdom; ^2^ EAG Laboratories Easton Maryland USA

## Abstract

Chlorothalonil is a fungicide which is highly toxic to aquatic organisms. However, in natural aquatic environments, it is very rapidly degraded, with a half‐life typically in hours, reducing exposure of aquatic organisms and the potential for effects. In standard regulatory studies looking at the chronic toxicity of chlorothalonil to fathead minnow, the most sensitive endpoint was fecundity. A standard fish full–life cycle study, where chlorothalonil concentrations were maintained constant throughout, resulted in a no‐observed‐effect concentration (NOEC) of 1.4 µg/L. Comparing peak modeled exposure concentrations to this NOEC can result in the chronic risk to fish being considered unacceptable. The present study investigated the effect on fecundity in fathead minnow using a fish short‐term reproduction assay. Five different exposure profiles were employed with time‐varying concentrations based on realistic worst‐case modeled environmental exposure profiles, multiplied by an assessment factor of 10, which resulted in maximum measured concentrations up to 15.5 µg/L. There were no effects on fecundity from any of the exposure profiles tested. Therefore, based on these more realistic exposure profiles, the chronic risk to fish could be considered acceptable if these exposures were deemed to be representative of the worst case. *Environ Toxicol Chem* 2019;38:1549–1559. © 2019 The Authors. *Environmental Toxicology and Chemistry* published by Wiley Periodicals, Inc. on behalf of SETAC.

## INTRODUCTION

Chlorothalonil is a nonsystemic foliar fungicide used globally since its introduction in 1966 in many agricultural crops and in noncrop uses such as turf. It is highly toxic to aquatic organisms, particularly fish, with acute 96‐h median lethal concentration (LC50) values typically in the region of 16 to 76 µg/L (European Commission 2006). Chronic toxicity has also been tested in a fathead minnow fish full–life cycle study lasting approximately 300 d, with quantitative endpoints including total number of eggs, eggs per brood, hatchability, survival, growth, and development, reporting a no‐observed‐effect concentration (NOEC) of 3 μg/L (European Commission 2006). Onduka et al. (2012) reported a NOEC from an early–life stage test with mummichog (*Fundulus heteroclitus*) embryos of 11 μg/L and a partial–life cycle study with sockeye salmon (*Oncorhynchus nerka*) reported effects of reduced alevin condition factor at both 5 and 0.5 µg/L (Du Gas et al. 2017).

These fish studies were conducted in the laboratory with chlorothalonil concentrations maintained throughout. However, chlorothalonil is very rapidly dissipated in natural aquatic environments, with half‐life (50% degradation time [DT50]) values of less than 1 d. While studying the acute toxicity of chlorothalonil to fish, Davies (1988) estimated a DT50 in an aerated natural water system containing fish and aufwuchs (surface coating comprising animal and plant life) of 4.4 h. Following regulatory review in the European Union, the agreed endpoint for dissipation in a laboratory water/sediment systems was a DT50 of 2.5 h (European Commission 2006). Dissipation is also rapid under field conditions, with DT50 values derived in aquatic microcosm studies of <1 d (European Commission 2016). This rapid dissipation and consequent reduction in exposure to chlorothalonil would be expected to reduce the potential for impacting aquatic organisms. Ernst et al. (1991) confirmed the reduction in the potential risk from chlorothalonil, following overspray of a natural pond containing caged rainbow trout. There was no mortality of the trout, despite initial chlorothalonil concentrations being 2 to 13 times above laboratory‐determined LC50 values. If dissipation is attributable to degradation, consideration needs to be given to the potential impact from any environmental metabolites; and this can be a significant part of the overall environmental risk assessment undertaken for registration of plant protection products. There are many environmental metabolites of chlorothalonil that have been tested for toxicity to aquatic organisms, confirming the reduction in toxicity and risk through degradation (European Commission 2006, 2016). Regardless, the focus of the present study was characterization of risk from the parent compound.

When it comes to assessing chronic risk to fish from chlorothalonil, including any sublethal effects on growth, development, or fecundity, the likelihood is that the rapid environmental dissipation will similarly reduce the potential risk. However, this reduced chronic risk is more difficult to demonstrate than acute because, unlike fish mortality, which is likely to be visible if it were to occur in the environment, effects on the sublethal endpoints are not so readily visible or quantifiable in the environment. Nevertheless, for a product to be used, it is necessary to be able to demonstrate acceptable chronic risk within the existing regulatory frameworks for plant protection products. For example, chronic risk assessments in the European Union (European Food Safety Authority 2013) and the United States (US Environmental Protection Agency 2017) compare the ratio of modeled exposure concentrations (predicted or estimated environmental concentrations) and effect endpoints (NOEC, no‐observed–adverse effect concentration, or concentration producing an *x*% effect), together with an appropriate assessment factor, to decide whether the risk is acceptable. Ideally, to predict the potential for effects from modeled exposure profiles, these profiles should be simulated in the effects studies (Brock et al. 2010). However, environmental exposures are often variable in both duration and magnitude of exposure, while in standard acute and chronic effects testing exposures are maintained. Most risk assessment schemes, through the use of assessment factors, are designed to be precautionary and protective at lower tiers, rather than predictive (Chapman et al. 1998; Posthuma et al. 2008). Consequently, the predicted or estimated environmental concentrations used in first‐tier assessment are the maximum values. At this first tier no account is taken of any time variable component in exposure, which can include losses through dissipation and repeat exposures from multiple events. If this first‐tier assessment does not result in acceptable risk, further evaluation can take any time‐varying exposures into account. The standard approach to incorporating time‐variable exposures has been to use a time‐weighted average exposure concentration in this chronic risk characterization. The US Environmental Protection Agency (2017) uses a 56‐ or 60‐d time‐weighted average exposure as a default for chronic risk to fish. The European Food Safety Authority's (2013) aquatic guidance proposes that a 7‐d time‐weighted average may be used as a default. However, this guidance goes on to describe situations when the use of a time‐weighted average approach in the assessment may not be appropriate. These situations are if the exposure was not maintained in the effects testing, the effects endpoint is based on a developmental process during a specific sensitive life stage that may last a short time only, mortality occurs early in the test, the acute to chronic ratio based on mortality is <10, or latency of effects has been demonstrated or is expected. Further to this, the European Food Safety Authority has more recently recommended that until it gives additional guidance, time‐weighted average approaches are unlikely to be sufficiently robust to be used in regulatory risk assessment (European Food Safety Authority 2015). Therefore, to incorporate time‐variable exposures into European Union risk assessment, a different approach is needed.

A second approach to considering effects from time‐variable exposures is to experimentally investigate effects following such exposures, rather than using the maintained exposures required in standard laboratory chronic toxicity testing (European Food Safety Authority 2013). There are a number of examples published in the literature of time‐varying or pulsed‐exposure studies with pesticides. These include studies on green algae (Vallotton et al. 2008; Brain et al. 2012a), aquatic macrophytes (Brain et al. 2012b; Boxall 2013), aquatic invertebrates (Naddy and Klaine 2001; Cold and Forbes 2004), and fish (Beyger et al. 2012).

The focus in the present study was on following the European aquatic guidance (European Food Safety Authority 2013) to characterize the risk of chlorothalonil to fish under more realistic, but worst‐case, time‐varying exposures, as part of the European Union registration package. The exposure was based on exposure profiles derived using the model from the Forum for the Co‐ordination of Pesticide Fate Models and Their Use (FOCUS), the European Union's surface water exposure model used in regulatory risk assessment. However, the approach in the present study is considered generally applicable to examine the chronic risk to fish and other organisms in situations where, because of rapid dissipation, realistic time‐variable exposures show a very different exposure profile from the maintained concentrations in standard studies.

## METHODS

### Selection of relevant chronic endpoints to study

Standard regulatory aquatic toxicity studies, including fish full–life cycle studies, are conducted with maintained exposures; and the resulting NOECs are used in the initial risk characterization. The NOEC used is the lowest based on the parameters measured, which include hatching, development, survival, growth, and reproduction. However, if the focus of the risk assessment requiring refinement is chronic risk to fish and the chosen approach is to experimentally examine the effect of exposure to short‐term pulses, it is not practical, nor indeed should it be necessary, to expose every part of the fish life cycle to the pulses and assess all chronic endpoints. Beyger et al. (2012) studied the chronic effects of a 4‐h pulsed exposure of endosulfan on Florida flagfish (*Jordanella floridae*), exposing larval fish (2–3 d posthatch) and monitoring their survival, growth, development, and reproduction over one full life cycle. Although there were significant effects on larval survival from the pulse at the highest concentration, there were no latent effects on life stages or phases which followed the pulsed exposure (e.g., reproduction). But it remained unclear whether a 4‐h pulsed exposure during the reproductive cycle would cause any significant effects. There was no justification given by Beyger for the pulse exposure being to the larval fish rather than during the reproductive phase. Although not every life stage needs to be investigated, it is necessary to justify which life stage(s) is exposed. This can be based on information regarding the sensitivity of different life stages to the chemical or, alternatively, on the timing of realistic environmental exposures and specific stages of the fish life cycle.

For chlorothalonil, the existing fish full–life cycle study with constant exposure was used to identify the most sensitive life stage and endpoint, from the parameters measured. The reported and agreed endpoint for use in risk assessment (European Commission 2006) from the chlorothalonil fish full–life cycle study of 3 µg/L was based on numbers of eggs/spawn and hatchability of the F1 fry. “Eggs per female per day” was not evaluated statistically in the original study. A subsequent reanalysis of the data from the fish full–life cycle study showed that the endpoint “eggs per female per day” was more sensitive, with a NOEC of 1.4 µg/L (US Environmental Protection Agency 2015); and this lower value was adopted as the new chronic endpoint for fish for use in the regulatory risk assessment (European Commission 2016).

Thus, numbers of eggs per female per day was identified as the most senstive parameter measured in the fish full–life cycle study, driving the NOEC. Therefore, a fish short‐term reproduction assay based on the Organisation for Economic Co‐operation and Development test 229 (Organisation for Economic Co‐operation and Development 2012), a study designed to look specifically at these reproductive parameters, was employed to investigate the effects of pulsed exposures.

### Selecting appropriate exposure regimes

In the European Union, FOCUS surface water modeling is used to produce exposure values for use in risk assesment. The modeling includes inputs from spray‐drift, runoff, and drainage and is used to produce realistic worst‐case exposure scenarios in various edge‐of‐field water bodies (ditches, streams, and ponds), relevant for use in each crop (FOCUS 2012). The FOCUS model was designed to give maximum predicted environmental concentrations for use in risk assessment, although FOCUS at steps 3 and 4 produces detailed time‐variable concentration profiles, with hourly time steps, for up to 16 mo. These are considered representative time‐variable exposure profiles for use in the assessment (European Food Safety Authority 2013). In accordance with the European Food Safety Authority aquatic guidance, the exposure profiles for chlorothalonil were examined for exceedance of the tier 1 regulatory acceptable concentration (RAC) for chronic risk to fish, the endpoint of concern. Defined at a Society of Environmental Chemistry and Toxicology expert workshop (Brock et al. 2010), the RAC is derived from the effects assessment endpoint (e.g., NOEC) and is expressed in terms of a permissible concentration in the environment. The RAC is then used directly in the risk assessment by comparing it to the appropriate environmental exposure estimate. For chronic risk to fish, the RAC is derived in the first tier of risk assessment by dividing the effects endpoint by an assessment factor of 10. Therefore, in the present case the chronic RAC for fish for chlorothalonil is 0.14 μg/L.

The assumption is that exposures below the RAC have no significance with respect to the risk assessment and only exceedences of the RAC of 0.14 µg/L are of concern. The Exposure Pattern Analysis Tool (EPAT; Wang et al. 2010) was used to examine these exposure profiles. The EPAT is an analysis tool rather than a modeling tool, extracting a number of key parameters with respect to the exposure profiles, including the number and maximum concentration of peaks exceeding the RAC, the area under the curve concentrations, the duration of any exceedances, and the interval between them, together with time‐weighted average concentrations. All of these parameters are key to developing the representative, worst‐case exposure scenarios used in the present study.

The FOCUS modeling outputs, including appropriate mitigation measures to reduce surface water exposure (i.e., no‐spray buffer zones and vegetative filter strips) from FOCUS step 4 values for all relevant scenarios, were analyzed. For applications to winter and spring cereals, there were 34 relevant scenarios. Taking into account the exposure characteristics including peak heights, width, frequency, and time between peaks, 5 different exposure scenarios were developed for use in the pulsed‐dose study. The developed exposure profiles were then multiplied by a factor of 10, to account for the fact that, in the European Union, an assessment factor of 10 is applied to the chronic NOEC to derive the chronic RAC. The nominal concentrations of the pulses are shown in Table 1, together with the mean measured concentrations of the pulses obtained during the present study. Significant efforts were made to characterize the exposure pulses throughout the present study using an appropriate anaytical method for chlorothalonil residues in water (extraction followed by gas chromatography‐mass spectrometry).

**Table 1:** Pulse treatments nominal and measured concentrations

Treatment	Pulse/time between pulses	Duration of pulse	Nominal concentration (µg/L)	Mean measured concentration (µg/L)^a^
Negative control	—	—	0.0	<LOQ
Solvent control	—	—	0.0	<LOQ
Treatment 1	Pulse 1	11 d	4	2.2
	6 d			
	Pulse 2	11 d	6.5^b^	3.1
Treatment 2	Pulse 1	26 h	13	10.7
Treatment 3	Pulse 1	16 h	16	12.9
	2 d			
	Pulse 2	6 h	4	2.2
	5 d			
	Pulse 3	16 h	18	10.1
Treatment 4	Pulse 1	6 h	4.5	3.0
	5 d			
	Pulse 2	16 h	16	10.1
	7 d			
	Pulse 3	16 h	5	2.2
Treatment 5	Pulse 1	24 h	8	5.7
	16 d			
	Pulse 2	20 h	21^b^	14.1

^a^ Mean measured concentrations were calculated only using samples collected during the pulse.

^b^ Nominal concentrations were set to target an achieved concentration of 4.0 µg/L for treatment 1, pulse 2 and 16 µg/L for treatment 5, pulse 2.

LOQ = level of quantitation.

A decision was made not to include a positive control or repeat the continuous exposure scenario,which had been conducted previously in regulatory studies. Importantly, with the exception of a maintained exposure regime, the present study followed the requirements of test 229 (Organisation for Economic Co‐operation and Development 2012). Organisation for Economic Co‐operation and Development guideline studies are internationally agreed and validated to provide sufficient sensitivity such that effects (where present) can be observed and repeated (if required). Furthermore, it is counter to the “3Rs” (replacement, reduction, and refinement of the use of animals in testing) to repeat vertebrate testing and/or include positive controls when conducting such validated regulatory studies.

### Test chemical and dosing

Chlorothalonil technical material (batch 691403, Chemical Abstracts Service number 1897‐45‐6, 99.3% w/w active ingredient; Syngenta) was used for preparing dosing solutions in dimethylformamide, such that the concentration of dimethylformamide during the pulses was 100 µL/L. The solvent control received 100 µL/L dimethylformamide throughout the exposure period, and the negative control received dilution water only.

### Test fish

Adult fathead minnow were used in the test, originally supplied by Osage Catfisheries, where they were bred and raised under laboratory conditions. The fish were housed in mixed cultures at the testing laboratory for an acclimation period of approximately 6 wk prior to the test and were approximately 6 mo old at the start of the pre‐exposure period. There were no mortalities during the 7 d prior to pre‐expoure, and the fish recieved no treatment for diseases during acclimation or the test. Twenty males and 20 females were weighed at the start of pre‐exposure and gave mean weights of 3.06 and 1.64 g, respectively. The range of individual weights of male and female fish at the start of the pre‐exposure period was within ±20% of the mean weight of each sex, as required by Organisation for Economic Co‐operation and Development test 229. The fish were fed during the present study with live brine shrimp (*Artemia*) and a commercial flake food one to 3 times daily.

### Dilution water, environmental and water quality parameters

Freshwater for culturing and testing was from a well located on the testing facility site (EAG Laboratories, Easton, MD, USA). The water was passed through a sand filter to remove particles greater than approximately 25 μm and pumped into a 37 800‐L storage tank, where the water was aerated with spray nozzles. Prior to use, the water was filtered to 0.45 μm to remove fine particles and passed through an ultraviolet sterilizer. During the 4‐wk period prior to testing, water characteristic values were as follows: specific conductance 321 to 367 µS/cm, hardness 144 to 148 mg/L as CaCO_3_, alkalinity 176 to 178 mg/L as CaCO_3_, pH 8.0 to 8.2, and total organic carbon <1 mg/L.

A photoperiod was provided of ambient laboratory light for 16 h light and 8 h dark with a 30‐min transition period. Light intensity was measured at the beginning of the pre‐exposure phase, weekly during the exposure period, and at termination of the exposure period in 5 indiscriminately selected locations within the environmental chamber using a SPER Scientific model 840006 C light meter. Temperature (target 25 ± 1 °C) was measured in each test vessel weekly using a digital (National Institute of Standards and Technology–traceable) thermometer. Temperature also was monitored continuously in one negative control test vessel using a validated environmental monitoring system (AmegaView Central Monitoring System). Dissolved oxygen concentrations and pH were measured in each test vessel weekly during the exposure period. Hardness, alkalinity, and specific conductance were measured in one replicate test vessel of the negative control and the highest concentration treatment group weekly during the test, with measurements alternating between replicates at each measurement interval. Dissolved oxygen concentrations were measured using a Thermo Orion Star A213 dissolved oxygen meter, and measurements of pH were made using a Thermo Orion Model Dual Star pH/ISE meter. Specific conductance was measured using a Thermo Orion Star A122 portable conductivity meter. Hardness and alkalinity were measured by titration. All parameters were within the test guideline recommendations (US Environmental Protection Agency 2009; Organisation for Economic Co‐operation and Development 2012).

### Exposure system

A continuous flow diluter delivered each test concentration and the negative and solvent controls to the test vessels, with 4 replicate vessels per treatment. Syringe pumps (Harvard Apparatus) delivered volumes of stock solution and dimethylformamide for the solvent control to individual mixing chambers prior to delivery to the test vessels. Each test vessel was a 19‐L glass vessel with an approximately 10‐L volume to overflow with a flow‐through of approximately 10 vessel volume replacements daily. In treatments that had multiple doses, toxicant flows were initiated 3 h prior to the time 0 time point for each pulse. Three spawning substrates or tiles were maintained in each test vessel during the exposure period. A tile consisted of an inverted semicircular section of polyvinyl chloride pipe, approximately 10 cm in length.

### Analytical sampling and analysis

Water samples were taken to characterize the pulsed exposures during dosing and periodically when no test substance was being delivered to the test vessels. Samples (100 mL) were collected from mid‐depth in the exposure vessels into glass jars, acidified with 0.10 mL acetic acid, and subsequently processed and analyzed immediately. Replicate samples were frozen in plastic jars and stored at approximately –20 °C as backups.

Samples were extracted twice with toluene, and the extracts were combined and analyzed using a Hewlett‐Packard Model 5975 gas chromatograph equipped with an Agilent model 7890 mass selective detector operated in the selected ion monitoring mode, using an Agilent HP‐5MS UI (30 m × 250 μm, 0.25 µm film thickness) column. The level of quantitation was set at 0.249 µg/L.

### Test procedure

The experimental design was based on the fish short‐term reproduction assay guidelines (OCSPP 890.1350, US Environmental Protection Agency 2009; test 229, Organisation for Economic Co‐operation and Development 2012). This exposes actively spawning females and sexually mature males for 21 d, with fecundity as one of the endpoints measured.

To ensure that fish were actively spawning at the start of the exposure, an 18‐d pre‐exposure period was conducted under control conditions. Two males with visible fatpad and tubercles and 4 females with a visible ovipositor were randomly assigned to each of 40 vessels. A breeding group was considered suitable for the test if there were at least 15 eggs/female/d in the vesssel and spawning occurred at least twice in the 7 d prior to exposure initiation. Following pre‐exposure, a total of 28 successfully spawning groups were assigned to treatments (4 replicates per treatment) using a stratified random procedure, intended to minimize variability in egg production between groups. Further details of the pre‐exposure performance of the groups used in the exposure phase are given in the Supplemental Data. The phase of the test during which the fish were exposed to pulses of chlorothalonil, followed by periods of no exposure, was 21 d for treatments 2 to 5. For treatment 1, where there were 30 d between the initial and final exposures to chlorothalonil, the fish were observed for a total of 36 d, as were the controls. The European Food Safety Authority aquatic guidance states that the duration of the test should be long enough to allow the observation of any delayed effects. Although this is almost always going to be a matter of judgment, it was considered by the authors that these exposure periods should be sufficient to show any effects, delayed or otherwise, on fecundity.

### Observations and measurements

Survival, fecundity, fertility, general behavior, and physiological observations of the fish were made daily. Any mortalities or external abnormalities (such as hemorrhage, discoloration) or behavioral changes were noted. Mortalities were recorded, and dead fish were removed from test vessels as soon as possible and discarded. Dead fish were not replaced in either the control or treatment test vessels.

The number of eggs spawned and the number of fertile eggs were determined for each breeding group daily during exposure. Spawning tiles were removed from the test chambers, and any eggs were counted. Clean tiles were added to the tanks to replace those removed. Individual spawning records were maintained for each of the 3 spawning tiles per replicate. Fecundity was expressed on the basis of eggs per surviving female per reproductive day per replicate and as cumulative eggs produced over the exposure period.

After eggs were counted on each spawning tile, the eggs were evaluated for fertilization success. A dye (toluidine blue) was applied to the eggs on the tile to highlight embryo development. The number of infertile eggs was counted, and the number of fertile eggs was calculated as the difference in the number of infertile eggs and the total number of eggs on the tile.

### Data analyses

Statistical analyses were performed to evaluate differences between treatment and control groups for survival, fecundity (eggs per female per reproductive day and cumulative number of eggs), and fertilization success ([fertile eggs/total eggs on tile] × 100).

**Figure 1:**
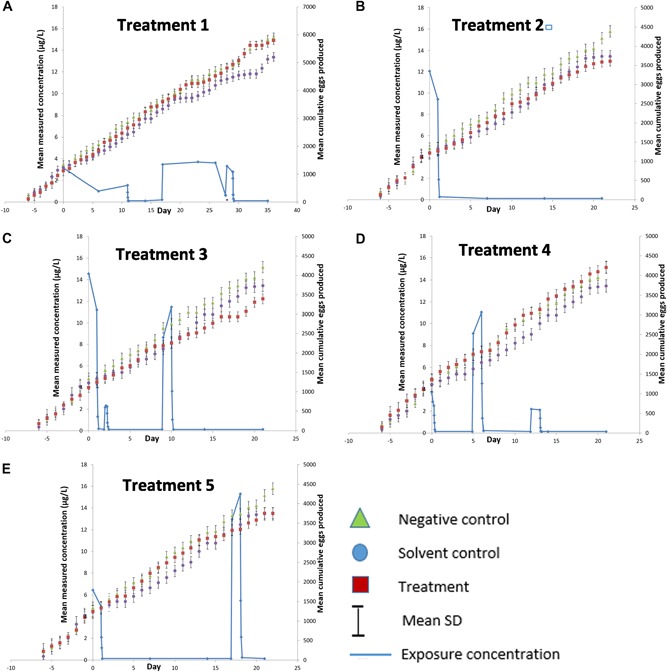
Graphical representation of cumulative egg production and pulse‐dosing treatments. SD = standard deviation.

No differences were detected between the 2 control groups (*t* test, *p* > 0.05) for any parameter. Therefore, the control data were pooled for comparisons among the treatment groups, as is commonly done in many regulatory studies. This is the so‐called protocol method, done in the belief that it increases the power to detect a treatment effect (Green 2014). However, other approaches are sometimes advocated, for example, the Organisation for Economic Co‐operation and Development (2006) generally recommends comparison with the solvent control, although stating that scientific judgment and regulatory guidance must be considered in deciding whether to pool nonsolvent and solvent controls. Others recommend comparison with the untreated control or both controls seperately. A discussion of these different approaches can be found in Green (2014). Regardless, the negative and solvent control data are presented in Tables 2 and 3 and in the Supplemental Data and plotted separately in Figure 1. All endpoints were also assessed for normality using the Shapiro‐Wilk test and for homogeneity of variance using Levene's test (α = 0.01, applied to residuals of analysis of variance). Following this, because the assumptions of normality and homogeneity of variance were met, all endpoints were analyzed using Dunnett's multiple comparison test to determine if treatment groups differed statistically from the control. Statistical tests used to evaluate treatment effects were performed at a confidence level of α = 0.05 with SAS 5 software.

**Table 2:** Effects of chlorothalonil on fathead minnow in a short‐term reproduction test for 21 d

	Percent survival to day 21	Cumulative number of eggs produced	Eggs per female per reproductive day	Percent fertility
Treatment^a^	(mean ± SD)	(mean ± SD)	(mean ± SD)	(mean ± SD)
Negative control	100 ± 0.0	2874 ± 253	34.2 ± 3.01	97.5 ± 1.2
Solvent control	100 ± 0.0	2503 ± 455	29.8 ± 5.41	96.9 ± 1.1
Pooled control	100 ± 0.0	2689 ± 394	32.0 ± 4.69	97.2 ± 1.1
Treatment 1	100 ± 0.0	3082 ± 250	36.7 ± 2.97	97.9 ± 1.2
Treatment 2	95.8 ± 8.4	2368 ± 636	29.3 ± 5.88	96.4 ± 1.7
Treatment 3	95.8 ± 8.4	2279 ± 455	27.5 ± 5.33	96.1 ± 1.8
Treatment 4	95.8 ± 8.4	2842 ± 1024	35.0 ± 11.0	96.5 ± 1.5
Treatment 5^b^	100 ± 0.0	2526 ± 370	28.7 ± 4.30	96.2 ± 1.1

^a^ There was no statistical difference between the negative and solvent controls; therefore, control data were pooled. There was no statistical difference in any treatment in comparison to the pooled control. Calculated using SAS System for Windows. Manual calculations may differ slightly.

^b^ Treatment 5 data are from termination on day 22.

SD = standard deviation.

**Table 3:** Effects of chlorothalonil on fathead minnow in a short‐term reproduction test for 36 d

	Percent survival to termination	Cumulative number of eggs produced	Eggs per female per reproductive day	Percent fertility
Treatment^a^	(mean ± SD)	(mean ± SD)	(mean ± SD)	(mean ± SD)
Negative control	95.8 ± 8.4	4564 ± 612	32.2 ± 3.99	95.9 ± 3.4
Solvent control	100 ± 0.0	3966 ± 763	27.5 ± 5.30	94.6 ± 3.1
Pooled control	97.9 ± 5.9	4265 ± 715	29.9 ± 5.01	97.1 ± 1.0
Treatment 1	100 ± 0.0	4684 ± 697	32.5 ± 4.87	97.6 ± 0.5

^a^ There was no statistical difference between the negative and solvent controls; therefore, control data were pooled. There was no statistical difference in any treatment in comparison to the pooled control. Calculated using SAS System for Windows. Manual calculations may differ slightly.

SD = standard deviation.

## RESULTS

### Environmental conditions

Test temperature measurements ranged from 24.8 to 26.8 °C, pH 7.9 to 8.3, dissolved oxygen 5.2 to 8.2 mg/L (>60% saturation), hardness 132 to 148 mg/L as CaCO_3_, conductivity 346 to 390 µS/cm, and light intensity 556 to 837 lux.

### Analytical data

The mean measured concentrations of the pulses and their duration are shown in Table 1. The actual measurements and the exposure profiles are given in Figure 1. Measured concentrations were below nominal, as might be expected for a compound so readily dissipated; but this had no impact on the outcome of the present study because it is the measured profiles which would be used for the risk characterization.

### Biological results

Mean percent survival, cumulative number of eggs produced, eggs per female per reproductive day, and percent fertility are shown in Tables 2 and 3. The data for individual replicates are given in the Supplemental Data.

The mean percent survival on day 21 in the pooled control, treatment 1, treatment 2, treatment 3, treatment 4, and treatment 5 was 100, 100, 95.8, 95.8, 95.8, and 100%, respectively. The mean percent survival on day 36 in the pooled control and treatment 1 was 97.9 and 100%, respectively. There were no statistically significant decreases in survival in any treatment group in comparison to the pooled control during the test according to Dunnett's test (*p* > 0.05).

The mean cumulative numbers of eggs produced over the 21‐d test in the negative control and solvent control groups were 2874 and 2503, respectively. The mean cumulative numbers of eggs produced over 21 d in the pooled control, treatment 1, treatment 2, treatment 3, treatment 4, and treatment 5 (22 d) were 2689, 3082, 2368, 2279, 2842, and 2526 eggs, respectively. Total numbers of eggs produced in treatments 1 through 5 represented 115, 88, 85, 106, and 94% of pooled control values, respectively. The mean cumulative numbers of eggs produced over 36 d in the pooled control and treatment 1 were 4265 and 4684, respectively, with the treatment 1 value being 110% of the pooled control. There were no statistically significant differences in egg production in any treatment groups in comparison to the pooled control according to Dunnett's test (*p* *>* 0.05). The mean cumulative numbers of eggs for the control, solvent control, and each treatment recorded daily throughout the present study are shown in Figure 1, along with the measured exposure concentrations for each treatment.

The mean numbers of eggs per female per reproductive day over 21 d in the pooled control, treatment 1, treatment 2, treatment 3, treatment 4, and treatment 5 were 32.0, 36.7, 29.3, 27.5, 35.0, and 28.7, respectively. Numbers of eggs per female per reproductive day in treatments 1 through 5 were 115, 92, 86, 109, and 90% of the pooled control value, respectively. The mean numbers of eggs per female per reproductive day over the 36 d in the pooled control and treatment 1 were 29.9 and 32.5, respectively, with the treatment 1 value being 109% of the pooled control. There was no statistically significant difference in egg production in treatments 1 through 5 in comparison to the pooled control according to Dunnett's test (*p* > 0.05).

The mean fertilization success rates over 21 d in the pooled control, treatment 1, treatment 2, treatment 3, treatment 4, and treatment 5 were 97.2, 97.9, 96.4, 96.1, 96.5, and 96.3%, respectively. The mean fertilization success rates over 36 d in the pooled control and treatment 1 were 97.1 and 97.6%, respectively. There were no statistically significant differences in percent fertilization success in any treatment groups in comparison to the pooled control according to Dunnett's test (*p* *>* 0.05).

There were no statistically significant effects on any of the parameters measured (survival, fecundity, and fertility) for any of the treatments. The most sensitive endpoint identified in the fish full–life cycle study with fathead was reproduction, and in the present study the different treatment means ranged from 85 to 115% of pooled control values for total number of eggs and eggs per female per day. Thus, although there was some variability between treatments and the controls, there was no evidence of any effect on these cumulative reproductive parameters.

## DISCUSSION

There were no significant effects on survival, fecundity, and fertility from any of the pulsed‐dose treatments. Test 229, the guideline on which the test procedure was based, acknowledges that quantification of egg production is variable and that the coefficient of variation may range from 20 to 60% (Organisation for Economic Co‐operation and Development 2012). The guideline goes on to say that at the higher range of the coefficient of variation the test would not be able to detect a significant decrease in egg production smaller than 70% and at the lower range a decrease of 40 to 50% could be detected with acceptable power (80%). As suggested by the guideline, the potential power of the test was maximized using a 4‐replicate design instead of 2, and the resultant coefficient of variations for egg production were low (<20%). Thus, the power of the present study was at the higher end of that predictable by a guideline‐compliant study. Regardless of statistical power, the cumulative number of eggs and eggs per female per day in the treatments ranged from 85 to 115% of the pooled controls. There was no evidence, therefore, of any treatment effects.

Based on the parameters measured, each treatment therefore represents a no‐observed‐effect level. Nevertheless, a question remains as to how these results should be expressed because the no‐observed‐effect level is not a concentration; it is an exposure profile. There is also the issue of how the results should be used in risk assessment. The aquatic guidance (European Food Safety Authority 2013) states that for long‐term refined exposure studies such as these, a chronic RAC can be derived, expressed in terms of the peak exposure concentration. In risk assessment, this RAC is then compared with peak modeled exposure concentrations. However, this approach only incorporates one parameter, the magnitude of a single pulse, while ignoring pulse duration, any other pulses, and the time between pulses. One way of expressing the result could be to express the exposure profile as a time‐weighted average concentration. However, it would be necessary to have, or assume, information about the toxicological independence, or otherwise, of exposures. Furthermore, concerns over the use of time‐weighted average approaches have recently been raised (European Food Safety Authority 2015), as discussed earlier (see *Introduction*). Clearly, in the absence of the acceptance of a time‐weighted average approach, a pulsed‐dose study cannot easily be used to generate a meaningful RAC because it is an exposure profile with changing concentration. What can be done, however, is to decide first whether the effects from such an exposure profile are acceptable and then whether the exposure profile, to quote the European Food Safety Authority (2013) guidance, is “realistic to worst‐case,” taking into account any assessment factor that might be applied.

One purpose of the present study is to show how pulsed‐dose effects data might be used, together with exposure data, in a risk assessment. For a compound with episodic, time‐variable exposures and rapid dissipation, the pragmatic way to address the chronic risk to fish is to look at the most sensitive life stage(s) and endpoint(s) or just at a specific life stage, if exposure is known to coincide only with that life stage. Consideration needs to be given as to whether there is a possibility that by focusing on, and addressing, a single endpoint other potential issues may be missed. However, it is likely that by addressing the most sensitive endpoint, together with worst‐case exposures, other endpoints will be covered from a risk‐assessment perspective. Chronic studies with fathead minnow have identified fecundity as the most sensitive endpoint following exposure to chlorothalonil, rather than hatching, survival, growth, or development (European Commission 2006, 2016). However, based on the environmental fate properties of chlorothalonil, exposures are likely to be acute (short‐term), so survival/mortality is another potential endpoint of concern. Both fecundity and survival endpoints were addressed in the present study, with no significant effects on either, even though the maximum exposure concentration was 15.5 µg/L, from a pulsed exposure that averaged 14.1 µg/L over 20 h. This compares to a reported 48‐h LC50 for chlorothalonil to fathead minnow of 22.9 µg/L (Sherrard et al. 2003) and a LOEC from a fathead minnow fish full–life cycle study of 3 µg/L, based on fecundity (European Commission 2016). What is clear is that, in the present study, all pulsed concentrations tested were far in excess of the fish chronic RAC of 0.14 µg/L used in the initial risk assessment in European Union (fish full–life cycle NOEC of 1.4 μg/L divided by an assessment factor of 10) and produced no significant adverse effects on fish fecundity.

Because there were no effects observed on the endpoints measured in the present study, there is no decision to be made about whether any effects were acceptable. However, this decision on acceptability could be a concern if the effects were, for example, short‐term and reversible. Another point raised by the European Food Safety Authority for chronic pulsed‐dose studies is that “the duration of the test should be long enough to allow the observation of any delayed effects.” This is difficult because there could be uncertainty over delayed effects or even potential for effects on subsequent generations, in the absence of a mechanistic explanation of effects. The observation periods for the different treatments were at least 3 wk after the initial exposures but as short as 3 d after the last exposures. However, given that spawning frequency was approximately every 3 d in the present study, we consider it long enough to pick up effects on fecundity.

**Figure 2:**
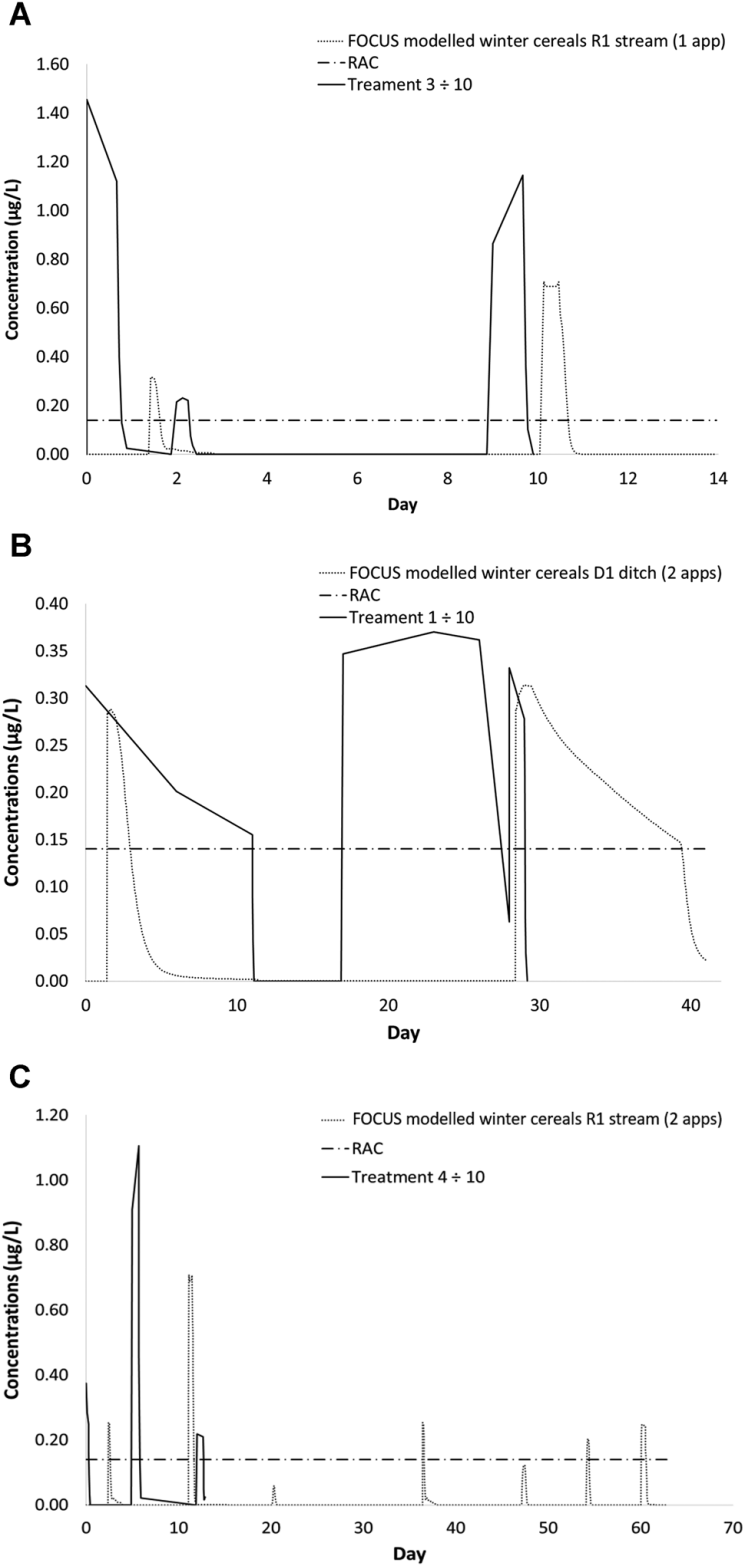
Example Forum for the Co‐ordination of Pesticide Fate Models and Their Use (FOCUS) outputs and pulse exposure profiles. RAC = regulatory acceptable concentration.

A further requirement for the risk assessment is to compare these tested exposure profiles and decide whether they are representative of environmental exposures or which potential environmental exposures they are representative of. For the European Union, this is done by comparison with modeled FOCUS exposure profiles for all relevant exposure scenarios based on crop, application rate, and timing. This comparison needs to take into account that in the European Union an assessment factor of 10 is applied to endpoints in chronic risk assessments by reducing the study exposure profile by a factor of 10 to compare with the modeled environmental exposure (as is done when producing a RAC from a constant exposure study). It is then something of a judgment call as to whether the study exposure profile is “realistic to worst‐case.” Examples of this comparison are given for 3 different scenarios for applications of chlorothalonil to winter wheat in Figure 2. It seems that the study exposure profile is realistic worst‐case for Figure 2A and 2B because these cover the number and magnitude of modeled exposure profiles. Furthermore, the time between exposures is, if anything, worse‐case because it is shorter in the test than in the model. However, for the exposure in Figure 2C, while the magnitude of exposures and the interval between them are clearly covered for the early exposures, there are some further minor exceedances of the RAC observed in the model, after a gap of approximately 30 d. This is where some judgment and pragmatism have to be applied. Given the time between the exceedances and the small magnitude of the later exceedances, it is not unrealistic to consider that these would not contribute significantly to any toxicity or effects, particularly because none were observed from all of the different exposure patterns tested. Explicit proof of toxicological independence would require further animal studies, and it is not considered that this is warranted. Indeed, if further testing was conducted to establish the toxicological independence of pulses, test concentrations or durations would likely have to be increased significantly to induce effects (because none were observed at 10 times the current modeled peak concentrations). The result of this would be unrealistic exposure in the study, which would in turn create a more complex interpretation for risk assessment and likely raise the severity classification of the experiment with regard to the fish (Hawkins et al. 2011). Another approach is to use toxicokinetic/toxicodynamic modeling to extrapolate to other exposure profiles and to explore the potential for organism‐level recovery between pulses (Jager et al. 2011; Ashauer et al. 2013). This can be an extremely valuable tool to address these time‐variable exposures in regulatory risk assessments, complementing the existing time‐weighted average and pulse‐dosed approaches (European Food Safety Authority 2018).

Unlike the time‐weighted average approach, this pulsed‐dose approach involves additional testing. Thus, ideally, if they can be shown to be protective, time‐weighted average approaches are preferable, requiring fewer resources and, in the case of vertebrates, minimizing animal testing. In the present study, the majority of the exposure scenarios were typified by short but often relatively high‐magnitude pulses, and perhaps the focus should be on acute exposure and effects. For chlorothalonil, most exposure scenarios passed the chronic risk assessment based on European Union conservative time‐weighted average approaches, with the maximum 7‐d time‐weighted average concentrations being below the tier 1 RAC of 0.14 µg/L. The worst‐case drainage scenarios, however, were typically 0.2 to 0.3 µg/L for up to 2 wk, resulting in a maximum 7‐d time‐weighted average exceeding the tier 1 RAC of 0.14 µg/L. Thus, use of the time‐weighted average would have allowed the conclusion of acceptable risk to be reached without this additional testing for many scenarios and reduced the number of scenarios required in such a pulsed‐dose approach. Use of a time‐weighted average approach in this way could, in turn, reduce animal use or alternatively allow more focused scenario testing depending on the environmental behavior of the compound in question.

The European Food Safety Authority (2013) guidance discusses that the time‐weighted average approach is based on reciprocity (Haber's law). This predicts that effects will be similar from exposure for a short time to a greater concentration compared with exposure for a longer time to a smaller concentration, with the European Food Safety Authority citing Giesy and Graney (1989). However, as discussed by Giesy and Graney, although for most classes of toxicants there seems to be a relationship between duration and intensity of dose, a curvilinear, hyperbolic relationship is the most common dose response, attributable to differences in damage and rate of repair, and linear reciprocity is not a typical dose response. Nevertheless, an expert meeting of the European Food Safety Authority and member state ecotoxicologists agreed that, until further guidance was available to test linear reciprocity and the latency of effects, time‐weighted average approaches are unlikely to be sufficiently robust to be used in regulatory risk assessment (European Food Safety Authority 2015). A subsequent expert meeting confirmed the view that linear reciprocity is the basis of the time‐weighted average approach and a prerequisite for its use in risk assessment (European Food Safety Authority 2016). Thus, if the time‐weighted average approach is to be accepted in the European Union, further work to demonstrate whether it is protective will be required; and until such time, alternative approaches in risk assessment, such as the present pulsed‐dose approach, will be necessary.

It is important to note that at the edge of field scale at which the risk assessment is done, the actual risk from chlorothalonil to aquatic environments will not have increased significantly over the last 50 yr or so since its introduction. In fact, quite the contrary, like many plant protection products, labeled use rates of chlorothalonil have declined significantly both in the European Union and in the United States, and mitigation measures have been introduced to reduce entry into aquatic environments and consequent exposure of aquatic environments (see, for example, US Environmental Protection Agency 1999). There is an argument that the newer guidance may now reflect risks which were not highlighted earlier. However, the situation remains that the real risk to fish is likely to have reduced whereas the perception of the magnitude of that risk has increased with changing guidance. The 2002 guidance for aquatic risk assessment (European Commission 2002) only required a long‐term/chronic test if the water phase DT50 from the water–sediment study was >2 d at an environmentally relevant pH (in the range 6–9). Chlorothalonil had a whole system DT50 <2 d in the water–sediment study with an agreed endpoint of 2.5 h (European Commission 2006). Therefore, following the 2002 guidance, it did not require chronic testing, leading to the conclusion that because there was no chronic exposure, there is low chronic risk. The review report (European Commission 2006) stated, “Given the rapid dissipation in aquatic systems, chronic exposure due to the agricultural applications is considered less likely.” The position “no chronic exposure equals low chronic risk” was generally accepted, with the focus on acute exposure and risk. However, the subsequent guidance (European Food Safety Authority 2013) requires an assessment for chronic risk, unless the DT90 attributable to hydrolysis is <1 d. Chlorothalonil does have a DT90 <1 d in most relevant aquatic systems. However, dissipation is generally driven by microbial and/or photodegradation rather than hydrolysis (Kwon and Armbrust 2006), and chlorothalonil therefore requires fish chronic testing and subsequent risk assessment in the European Union, following current guidance. Acceptable risk cannot be demonstrated using the existing fish full–life cycle study where the concentrations were maintained; thus, further refinement taking into account more realistic time‐variable exposures is required. In the absence of time‐weighted average approaches, the pulsed‐dose approach adopted in the present study is seen as an appropriate way to better characterize the chronic risk.

## CONCLUSION

For chlorothalonil, a pulsed‐dose study looking at fecundity, the most sensitive endpoint derived from a fish full–life cycle study, is an appropriate way to examine the potential for adverse chronic effects from time‐varying exposures. However, for risk assessment, consideration needs to be given as to whether those exposures are realistic worst‐case. The present study has highlighted the importance of examining the available effects data, to select the most appropriate endpoint for investigation, and understanding the environmental fate profile, to best characterize the risk. The approach in the present study could be applicable to risk assessment of other chemicals with similar exposure patterns and potential for adverse effects on fish and, indeed, other organisms. This can be seen as an alternative to time‐weighted average approaches or as a higher tier, if time‐weighted average approaches fail to demonstrate acceptable risk.

## Supplemental Data

The Supplemental Data are available on the Wiley Online Library at DOI: 10.1002/etc.4421.

## Disclaimer

The present study was funded by Syngenta, Oxon Italia, and Arysta LifeScience, European Union notifiers for chlorothalonil.

## Data Accessibility

Data, associated metadata, and calculation tools are available from the corresponding author (mick.hamer@syngenta.com).

## Supporting information

This article contains online‐only Supplemental Data.

Supporting information.
