# Supplementary material for: A Pulsed‐Dose Study Evaluating Chronic Toxicity of Chlorothalonil to Fish: A Case Study for Environmental Risk Assessment
Source: Environ Toxicol Chem. 2019 Jun 19;38(7):1549–59. doi: 10.1002/etc.4421 (PMC6851820; doi:10.1002/etc.4421)
Supplement: Supplementary file 1 — Supporting information. [file ETC-38-1549-s001.doc]

**Supporting Information**

**A Pulsed Dose study evaluating chronic toxicity of chlorothalonil to fish; A CASE STUDY For environmental Risk assessment**

Mick Hamer, Samuel K. Maynard, Suzanne Schneider

Table 1 Survival of Fathead Minnows During the 36-Day Exposure to Chlorothalonil

| Treatment | Repl | Initial  No. of  Organisms ^1^ | Number Surviving by Day of Exposure | | | | | | | | | | | | |
| --- | --- | --- | --- | --- | --- | --- | --- | --- | --- | --- | --- | --- | --- | --- | --- |
|  |  |  | 0 | 1 | 2 | 3 | 4 | 5 | 6 | 7 | 8 | 9 | 10 | 11 | 12 |
| Negative | A | 6 | 6 | 6 | 6 | 6 | 6 | 6 | 6 | 6 | 6 | 6 | 6 | 6 | 6 |
| Control | B | 6 | 6 | 6 | 6 | 6 | 6 | 6 | 6 | 6 | 6 | 6 | 6 | 6^2^ | 6 ^2^ |
|  | C | 6 | 6 | 6 | 6 | 6 | 6 | 6 | 6 | 6 | 6 | 6 | 6 | 6 | 6 |
|  | D | 6 | 6 | 6 | 6 | 6 | 6 | 6 | 6 | 6 | 6 | 6 | 6 | 6 | 6 |
|  |  |  |  |  |  |  |  |  |  |  |  |  |  |  |  |
| Solvent | A | 6 | 6 | 6 | 6 | 6 | 6 | 6 | 6 | 6 | 6 | 6 | 6 | 6 | 6 |
| Control | B | 6 | 6 | 6 | 6 | 6 | 6 | 6 | 6 | 6 | 6 | 6 | 6 | 6 | 6 |
|  | C | 6 | 6 | 6 | 6 | 6 | 6 | 6 | 6 | 6 | 6 | 6 | 6 | 6 | 6 |
|  | D | 6 | 6 | 6 | 6 | 6 | 6 | 6 | 6 | 6 | 6 | 6 | 6 | 6 | 6 |
|  |  |  |  |  |  |  |  |  |  |  |  |  |  |  |  |
| Treatment 1 | A | 6 | 6 | 6 | 6 | 6 | 6 | 6 | 6 | 6 | 6 | 6 | 6 | 6 | 6 |
|  | B | 6 | 6 | 6 | 6 | 6 | 6 | 6 | 6 | 6 | 6 | 6 | 6 | 6 | 6 |
|  | C | 6 | 6 | 6 | 6 | 6 | 6 | 6 | 6 | 6 | 6 | 6 | 6 | 6 | 6 |
|  | D | 6 | 6 | 6 | 6 | 6 | 6 | 6 | 6 | 6 | 6 | 6 | 6 | 6 | 6 |
|  |  |  |  |  |  |  |  |  |  |  |  |  |  |  |  |
| Treatment 2 | A | 6 | 6 | 6 | 6 | 6 | 6 | 5 ^3^ | 5 | 5 | 5 | 5 | 5 | 5 | 5 |
|  | B | 6 | 6 | 6 | 6 | 6 | 6 | 6 | 6 | 6 | 6 | 6 | 6 | 6 | 6 |
|  | C | 6 | 6 | 6 | 6 | 6 | 6 | 6 | 6 | 6 | 6 | 6 | 6 | 6 | 6 |
|  | D | 6 | 6 | 6 | 6 | 6 | 6 | 6 | 6 | 6 | 6 | 6 | 6 | 6 | 6 |
| 1. Each replicate contained one spawning group consisting of four females and two males. 2. One female was noted to have the superior portion of tail lost. 3. One female died on Day 5. | | | | | | | | | | | | | | | |

**Table 1 continued Survival of Fathead Minnows During the 36-Day Exposure to Chlorothalonil**

| Treatment | Rep | Initial  No. of  Organisms ^1^ | Number Surviving by Day of Exposure | | | | | | | | | | | | |
| --- | --- | --- | --- | --- | --- | --- | --- | --- | --- | --- | --- | --- | --- | --- | --- |
|  |  |  | 0 | 1 | 2 | 3 | 4 | 5 | 6 | 7 | 8 | 9 | 10 | 11 | 12 |
| Treatment 3 | A | 6 | 6 | 6 | 6 | 6 | 6 | 6 | 6 | 6 | 6 | 6 | 6 | 6 | 6 |
|  | B | 6 | 6 | 6 | 6 | 6 | 6 | 6 | 6 | 6 | 6 | 6 | 6 | 6 | 6 |
|  | C | 6 | 6 | 6 | 6 | 6 | 6 | 6 | 6 | 6 | 6 | 6 | 6 ^3^ | 6 ^3^ | 6 ^3^ |
|  | D | 6 | 6 | 6 | 6 | 6 ^2^ | 6 | 6 | 6 | 6 ^2^ | 6 ^2^ | 6 ^2^ | 6 ^2^ | 6 ^2^ | 6 ^2^ |
|  |  |  |  |  |  |  |  |  |  |  |  |  |  |  |  |
| Treatment 4 | A | 6 | 6 | 6 | 6 | 6 | 6 | 6 | 6 | 6 | 6 | 6 | 6 | 6 | 6 |
|  | B | 6 | 6 | 6 | 6 | 6 | 6 | 6 | 6 | 6 | 6 | 6 | 6 | 6 | 6 |
|  | C | 6 | 6 | 6 | 6 | 6 | 6 | 6 | 6 | 6 | 6 | 6 | 6 | 6 | 6 |
|  | D | 6 | 6 | 6 | 6 | 6 | 6 | 6^4^ | 6^4^ | 5^5^ | 5 | 5 | 5 | 5 | 5 |
|  |  |  |  |  |  |  |  |  |  |  |  |  |  |  |  |
| Treatment 5 | A | 6 | 6 | 6 | 6 | 6 | 6 | 6 | 6 | 6 | 6 | 6 | 6 | 6 | 6 |
|  | B | 6 | 6 | 6 | 6 | 6 | 6 | 6 | 6 | 6 | 6 | 6 | 6 | 6 ^2^ | 6 ^2^ |
|  | C | 6 | 6 | 6 | 6 | 6 | 6 | 6 | 6 | 6 | 6 | 6 | 6 | 6 | 6 |
|  | D | 6 | 6 | 6 | 6 | 6 | 6 | 6 | 6 | 6 | 6 | 6 | 6 | 6 | 6 |
|  |  |  |  |  |  |  |  |  |  |  |  |  |  |  |  |
| 1. Each replicate contained one spawning group consisting of four females and two males. 2. One female noted to have black spots on abdomen. 3. One female was noted to have a red, swollen ovipositor 4. One female was noted to have a haemorrhaged ovipositor. 5. One female died on Day 7. | | | | | | | | | | | | | | | |

**Table 1 continued Survival of Fathead Minnows During the 36-Day Exposure to Chlorothalonil**

| Treatment | Rep | Initial  No. of  Organisms ^1^ | Number Surviving by Day of Exposure | | | | | | | | | Replicate  % Survival to  Day 21 | Treatment  % Survival to  Day 21 |
| --- | --- | --- | --- | --- | --- | --- | --- | --- | --- | --- | --- | --- | --- |
|  |  |  | 13 | 14 | 15 | 16 | 17 | 18 | 19 | 20 | 21 |  |  |
| Negative | A | 6 | 6 | 6 | 6 | 6 | 6 | 6 | 6 | 6 | 6 | 100 | 100 |
| Control | B | 6 | 6 ^2^ | 6 ^2^ | 6 ^2^ | 6 ^2^ | 6 ^2^ | 6 ^2^ | 6 ^2^ | 6 ^2^ | 6 ^2^ | 100 |  |
|  | C | 6 | 6 | 6 | 6 | 6 | 6 | 6 | 6 | 6 | 6 | 100 |  |
|  | D | 6 | 6 | 6 | 6 | 6 | 6 | 6 | 6 | 6 | 6 | 100 |  |
|  |  |  |  |  |  |  |  |  |  |  |  |  |  |
| Solvent | A | 6 | 6 | 6 | 6 | 6 | 6 | 6 | 6 | 6 | 6 | 100 | 100 |
| Control | B | 6 | 6 | 6 | 6 | 6 | 6 | 6 | 6 | 6 | 6 | 100 |  |
|  | C | 6 | 6 | 6 | 6 | 6 | 6 | 6 | 6 | 6 | 6 | 100 |  |
|  | D | 6 | 6 | 6 | 6 | 6 | 6 | 6 | 6 | 6 | 6 | 100 |  |
|  |  |  |  |  |  |  |  |  |  |  |  |  |  |
| Treatment 1 | A | 6 | 6 | 6 | 6 | 6 | 6 | 6 | 6 | 6 | 6 | 100 | 100 |
|  | B | 6 | 6 | 6 | 6 | 6 | 6 | 6 | 6 | 6 | 6 | 100 |  |
|  | C | 6 | 6 | 6 | 6 | 6 | 6 | 6 | 6 | 6 | 6 | 100 |  |
|  | D | 6 | 6 | 6 | 6 | 6 | 6 | 6 | 6 | 6 | 6 | 100 |  |
|  |  |  |  |  |  |  |  |  |  |  |  |  |  |
| Treatment 2 | A | 6 | 5 | 5 | 5 | 5 | 5 | 5 | 5 | 5 | 5 | 83.3 | 95.8 |
|  | B | 6 | 6 | 6 | 6 | 6 | 6 | 6 | 6 | 6 | 6 | 100 |  |
|  | C | 6 | 6 | 6 | 6 | 6 | 6 | 6 | 6 | 6 | 6 | 100 |  |
|  | D | 6 | 6 | 6 | 6 | 6 | 6 | 6 | 6 | 6 ^3^ | 6 ^3^ | 100 |  |
| 1. Each replicate contained one spawning group consisting of four females and two males. 2. One female was noted to have the superior portion of tail lost. 3. One male noted to have discoloration/scarring on fatpad. | | | | | | | | | | | | | |

**Table 1 continued Survival of Fathead Minnows During the 36-Day Exposure to Chlorothalonil**

| Treatment | Rep | Initial  No. of  Organisms ^1^ | Number Surviving by Day of Exposure | | | | | | | | | Replicate  % Survival to  Day 21 | Treatment  % Survival to  Day 21 |
| --- | --- | --- | --- | --- | --- | --- | --- | --- | --- | --- | --- | --- | --- |
|  |  |  | 13 | 14 | 15 | 16 | 17 | 18 | 19 | 20 | 21 |  |  |
| Treatment 3 | A | 6 | 6 | 6 | 6 | 6 | 6 | 6 | 6 | 6 | 6 | 100 | 95.8 |
|  | B | 6 | 6 | 6 | 6 | 6 | 6 | 6 | 6 | 6 | 6 | 100 |  |
|  | C | 6 | 6 ^3^ | 6 | 6 | 6 | 5 ^4^ | 5 | 5 | 5 | 5 | 83.3 |  |
|  | D | 6 | 6 ^2^ | 6 | 6 | 6 | 6 | 6 | 6 | 6 | 6 | 100 |  |
|  |  |  |  |  |  |  |  |  |  |  |  |  |  |
| Treatment 4 | A | 6 | 6 | 6 | 6 | 6 | 6 | 6 | 6 | 6 | 6 | 100 | 95.8 |
|  | B | 6 | 6 | 6 | 6 | 6 | 6 | 6 | 6 | 6 | 6 | 100 |  |
|  | C | 6 | 6 | 6 | 6 | 6 | 6 | 6 | 6 | 6 | 6 | 100 |  |
|  | D | 6 | 5 | 5 ^3^ | 5 ^3^ | 5 ^3^ | 5 | 5 | 5 | 5 ^2^ | 5 ^2^ | 83.3 |  |
|  |  |  |  |  |  |  |  |  |  |  |  |  |  |
| Treatment 5 ^5^ | A | 6 | 6 | 6 | 6 | 6 | 6 | 6 | 6 | 6 | 6 | 100 | 100 |
|  | B | 6 | 6 ^2^ | 6 ^2^ | 6 ^2^ | 6 ^2^ | 6 ^2^ | 6 | 6 | 6 | 6 | 100 |  |
|  | C | 6 | 6 | 6 | 6 | 6 | 6 | 6 | 6 | 6 | 6 | 100 |  |
|  | D | 6 | 6 | 6 | 6 | 6 | 6 | 6 | 6 | 6 | 6 | 100 |  |
|  |  |  |  |  |  |  |  |  |  |  |  |  |  |
| 1. Each replicate contained one spawning group consisting of four females and two males. 2. One female noted to have black spot on abdomen. 3. One female was noted to have a red, swollen ovipositor 4. One female died on Day 17. 5. Survival is presented from Day 22 when treatment was terminated. | | | | | | | | | | | | | |

**Table 1 continued Survival of Fathead Minnows During the 36-Day Exposure to Chlorothalonil**

| Treatment | Rep | Initial  No. of  Organisms ^1^ | Number Surviving by Day of Exposure | | | | | | | | |
| --- | --- | --- | --- | --- | --- | --- | --- | --- | --- | --- | --- |
|  |  |  | 22 | 23 | 24 | 25 | 26 | 27 | 28 | 29 | 30 |
| Negative | A | 6 | 6 | 6 | 6 | 6 | 6 | 6 | 6 | 6 | 6 |
| Control | B | 6 | 6 ^2^ | 6 ^2^ | 6 ^2^ | 6 ^2,3^ | 6 ^2,3,4^ | 5 ^5^ | 5 | 5 | 5 |
|  | C | 6 | 6 | 6 | 6 | 6 | 6 | 6 | 6 | 6 | 6 |
|  | D | 6 | 6 | 6 | 6 | 6 | 6 | 6 | 6 | 6 | 6 |
|  |  |  |  |  |  |  |  |  |  |  |  |
| Solvent | A | 6 | 6 | 6 | 6 | 6 | 6 | 6 | 6 | 6 | 6 |
| Control | B | 6 | 6 | 6 | 6 | 6 | 6 | 6 | 6 | 6 | 6 |
|  | C | 6 | 6 | 6 | 6 | 6 | 6 | 6 | 6 | 6 | 6 |
|  | D | 6 | 6 | 6 | 6 | 6 | 6 | 6 | 6 | 6 | 6 |
|  |  |  |  |  |  |  |  |  |  |  |  |
| Treatment 1 | A | 6 | 6 | 6 | 6 | 6 | 6 | 6 | 6 | 6 | 6 |
|  | B | 6 | 6 | 6 | 6 | 6 | 6 | 6 | 6 | 6 | 6 |
|  | C | 6 | 6 | 6 | 6 | 6 | 6 | 6 | 6 | 6 | 6 |
|  | D | 6 | 6 | 6 | 6 | 6 | 6 | 6 | 6 | 6 | 6 |
| 1. Each replicate contained one spawning group consisting of four females and two males. 2. One female was noted to have the superior portion of tail lost/haemorrhaging. 3. Female fish has slight amount of haemorrhaging on left side of caudal peduncle. 4. Female fish exhibiting loss of equilibrium 5. One female died on Day 27. | | | | | | | | | | | |

**Table 1 continued Survival of Fathead Minnows During the 36-Day Exposure to Chlorothalonil**

| Treatment | Rep | Initial  No. of  Organisms ^1^ | Number Surviving by Day of Exposure | | | | | | Replicate  % Survival to  Day 36 | | Treatment  % Survival to  Day 36 | |  |
| --- | --- | --- | --- | --- | --- | --- | --- | --- | --- | --- | --- | --- | --- |
|  |  |  | 31 | 32 | 33 | 35 | 36 |  | |  | | | |
| Negative | A | 6 | 6 | 6 | 6 | 6 | 6 | 100 | | 95.8 | |  | |
| Control | B | 6 | 5 | 5 | 5 | 5 | 5 | 83.3 | |  | |  | |
|  | C | 6 | 6 | 6 | 6 | 6 | 6 | 100 | |  | |  | |
|  | D | 6 | 6 | 6 | 6 | 6 | 6 | 100 | |  | |  | |
|  |  |  |  |  |  |  |  |  | |  | |  | |
| Solvent | A | 6 | 6 | 6 | 6 | 6^2^ | 6^2^ | 100 | | 100 | |  | |
| Control | B | 6 | 6 | 6 | 6 | 6 | 6 | 100 | |  | |  | |
|  | C | 6 | 6 | 6 | 6 | 6 | 6 | 100 | |  | |  | |
|  | D | 6 | 6 | 6 | 6 | 6 | 6 | 100 | |  | |  | |
|  |  |  |  |  |  |  |  |  | |  | |  | |
| Treatment 1 | A | 6 | 6 | 6 | 6 | 6 | 6 | 100 | | 100 | |  | |
|  | B | 6 | 6 | 6 | 6 | 6 | 6 | 100 | |  | |  | |
|  | C | 6 | 6 | 6 | 6 | 6 | 6 | 100 | |  | |  | |
|  | D | 6 | 6 | 6 | 6 | 6 | 6 | 100 | |  | |  | |
| 1. Each replicate contained one spawning group consisting of four females and two males. 2. One female noted to have a black spot on abdomen. | | | | | | | | | | | |  | |

Table 2 Survival, Fecundity and Ranking of Breeding Groups from the Pre-Exposure Period

| Ranking ^1^ | Tank  Number ^2^ | Percent Survival  to Day 18 | Cumulative  No. of Eggs  Produced  to Day 18 | Eggs per Female Reproductive  Day | Replicate Assignment  for Exposure ^1^ | |
| --- | --- | --- | --- | --- | --- | --- |
|  |  |  |  |  | Treatment | Replicate |
| 1 | 30 | 100 | 4626 | 64.3 | Negative Control | A |
| 2 | 22 | 100 | 4532 | 62.9 | Treatment 2 | A |
| 3 | 36 | 100 | 4210 | 58.5 | Treatment 4 | A |
| 4 | 23 | 100 | 4210 | 58.5 | Treatment 5 | A |
| 5 | 29 | 100 | 4168 | 57.9 | Treatment 1 | A |
| 6 | 39 | 100 | 4107 | 57.0 | Solvent Control | A |
| 7 | 11 | 100 | 4071 | 56.5 | Treatment 3 | A |
| 8 | 10 | 100 | 3978 | 55.3 | Treatment 4 | B |
| 9 | 27 | 100 | 3912 | 54.3 | Treatment 2 | B |
| 10 | 2 | 100 | 3898 | 54.1 | Solvent Control | B |
| 11 | 14 | 100 | 3856 | 53.6 | Treatment 5 | B |
| 12 | 20 | 100 | 3831 | 53.2 | Negative Control | B |
| 13 | 35 | 100 | 3821 | 53.1 | Treatment 1 | B |
| 14 | 19 | 100 | 3756 | 52.2 | Treatment 3 | B |
| 15 | 3 | 100 | 3673 | 51.0 | Treatment 3 | C |
| 16 | 40 | 100 | 3659 | 50.8 | Solvent Control | C |
| 17 | 18 | 100 | 3642 | 50.6 | Treatment 4 | C |
| 18 | 31 | 100 | 3597 | 50.0 | Treatment 1 | C |
| 19 | 7 | 100 | 3554 | 49.4 | Negative Control | C |
| 20 | 15 | 100 | 3542 | 49.2 | Treatment 2 | C |
| 21 | 5 | 100 | 3529 | 49.0 | Treatment 5 | C |
| 22 | 37 | 100 | 3430 | 47.6 | Treatment 2 | D |
| 23 | 8 | 100 | 3426 | 47.6 | Treatment 4 | D |
| 24 | 13 | 100 | 3378 | 46.9 | Negative Control | D |
| 25 | 32 | 100 | 3348 | 46.5 | Treatment 1 | D |
| 26 | 33 | 100 | 3064 | 42.6 | Treatment 3 | D |
| 27 | 9 | 100 | 3043 | 42.3 | Solvent Control | D |
| 28 | 28 | 100 | 3034 | 42.1 | Treatment 5 | D |
| ^1^ 36 spawning groups were ranked according to egg production (eggs per female reproductive day) and the top 28 groups were selected. Of the 28 groups, the top seven performers were randomly assigned to one replicate exposure chamber of each treatment or control group, then the next seven performers were assigned to one replicate of each group, and so on.  ^2^ 40 tanks were maintained during the pre-exposure period. Each tank contained one spawning group consisting of four females and two males. Four tanks were excluded due to mortality or health of fish. | | | | | | |

Table 3 Fecundity and Fertility of Fathead Minnows During the Pulse Dose Exposure to Chlorothalonil

| Day | Negative Control | | | | | | | | | | |
| --- | --- | --- | --- | --- | --- | --- | --- | --- | --- | --- | --- |
|  | Fecundity and Fertility by Replicate and Day of Exposure | | | | | | | | | | |
|  | Replicate A | |  | Replicate B | |  | Replicate C | |  | Replicate D | |
|  | Eggs  Produced ^1^ | Fertile  Eggs |  | Eggs  Produced ^1^ | Fertile  Eggs |  | Eggs  Produced ^1^ | Fertile  Eggs |  | Eggs  Produced ^1^ | Fertile  Eggs |
| 1 | 0 | 0 |  | 6 | 6 |  | 6 | 5 |  | 0 | 0 |
| 2 | 290 | 281 |  | 209 | 191 |  | 172 | 167 |  | 178 | 162 |
| 3 | 124 | 122 |  | 171 | 171 |  | 240 | 240 |  | 0 | 0 |
| 4 | 0 | 0 |  | 216 | 214 |  | 292 | 291 |  | 210 | 210 |
| 5 | 158 | 153 |  | 129 | 124 |  | 0 | 0 |  | 148 | 147 |
| 6 | 174 | 174 |  | 0 | 0 |  | 209 | 204 |  | 0 | 0 |
| 7 | 242 | 223 |  | 0 | 0 |  | 75 | 71 |  | 0 | 0 |
| 8 | 0 | 0 |  | 252 | 248 |  | 182 | 181 |  | 356 | 341 |
| 9 | 279 | 260 |  | 312 | 312 |  | 132 | 129 |  | 478 | 468 |
| 10 | 0 | 0 |  | 144 | 123 |  | 281 | 277 |  | 0 | 0 |
| 11 | 6 | 6 |  | 0 | 0 |  | 0 | 0 |  | 471 | 469 |
| 12 | 233 | 225 |  | 247 | 239 |  | 119 | 118 |  | 124 | 120 |
| 13 | 0 | 0 |  | 0 | 0 |  | 0 | 0 |  | 6 | 3 |
| 14 | 0 | 0 |  | 263 | 255 |  | 582 | 582 |  | 0 | 0 |
| 15 | 0 | 0 |  | 0 | 0 |  | 0 | 0 |  | 113 | 85 |
| 16 | 503 | 488 |  | 352 | 351 |  | 140 | 140 |  | 0 | 0 |
| 17 | 172 | 172 |  | 134 | 134 |  | 164 | 164 |  | 130 | 128 |
| 18 | 0 | 0 |  | 0 | 0 |  | 168 | 168 |  | 0 | 0 |
| 19 | 183 | 178 |  | 0 | 0 |  | 0 | 0 |  | 505 | 503 |
| 20 | 167 | 157 |  | 0 | 0 |  | 0 | 0 |  | 0 | 0 |
| 21 | 428 | 420 |  | 192 | 186 |  | 429 | 429 |  | 0 | 0 |
| ^1^ Egg production is presented as the total number of eggs present on three spawning tiles per replicate. | | | | | | | | | | | |

Table 3 continued Fecundity and Fertility of Fathead Minnows During the Pulse Dose Exposure to Chlorothalonil

| Day | Negative Control | | | | | | | | | | |
| --- | --- | --- | --- | --- | --- | --- | --- | --- | --- | --- | --- |
|  | Fecundity and Fertility by Replicate and Day of Exposure | | | | | | | | | | |
|  | Replicate A | |  | Replicate B | |  | Replicate C | |  | Replicate D | |
|  | Eggs  Produced ^1^ | Fertile  Eggs |  | Eggs  Produced ^1^ | Fertile  Eggs |  | Eggs  Produced ^1^ | Fertile  Eggs |  | Eggs  Produced ^1^ | Fertile  Eggs |
| 22 | 178 | 166 |  | 306 | 300 |  | 4 | 4 |  | 230 | 224 |
| 23 | 0 | 0 |  | 0 | 0 |  | 0 | 0 |  | 0 | 0 |
| 24 | 6 | 5 |  | 0 | 0 |  | 0 | 0 |  | 83 | 75 |
| 25 | 0 | 0 |  | 280 | 276 |  | 263 | 257 |  | 197 | 181 |
| 26 | 202 | 199 |  | 0 | 0 |  | 152 | 132 |  | 103 | 103 |
| 27 | 116 | 112 |  | 189 | 180 |  | 0 | 0 |  | 0 | 0 |
| 28 | 193 | 186 |  | 0 | 0 |  | 0 | 0 |  | 0 | 0 |
| 29 | 207 | 206 |  | 0 | 0 |  | 0 | 0 |  | 0 | 0 |
| 30 | 0 | 0 |  | 0 | 0 |  | 273 | 261 |  | 179 | 177 |
| 31 | 387 | 377 |  | 0 | 0 |  | 609 | 608 |  | 426 | 422 |
| 32 | 0 | 0 |  | 515 | 508 |  | 0 | 0 |  | 17 | 16 |
| 33 | 451 | 448 |  | 22 | 21 |  | 0 | 0 |  | 0 | 0 |
| 34 | 202 | 201 |  | 0 | 0 |  | 0 | 0 |  | 0 | 0 |
| 35 | 435 | 427 |  | 0 | 0 |  | 256 | 243 |  | 0 | 0 |
| 36 | 0 | 0 |  | 277 | 270 |  | 0 | 0 |  | 0 | 0 |
| Total: | 5336 | 5186 |  | 4216 | 4109 |  | 4748 | 4671 |  | 3954 | 3834 |
| ^1^ Egg production is presented as the total number of eggs present on three spawning tiles per replicate. | | | | | | | | | | | |

Table 3 continued Fecundity and Fertility of Fathead Minnows During the Pulse Dose Exposure to Chlorothalonil (continued)

| Day | Solvent Control | | | | | | | | | | |
| --- | --- | --- | --- | --- | --- | --- | --- | --- | --- | --- | --- |
|  | Fecundity and Fertility by Replicate and Day of Exposure | | | | | | | | | | |
|  | Replicate A | |  | Replicate B | |  | Replicate C | |  | Replicate D | |
|  | Eggs  Produced ^1^ | Fertile  Eggs |  | Eggs  Produced ^1^ | Fertile  Eggs |  | Eggs  Produced ^1^ | Fertile  Eggs |  | Eggs  Produced ^1^ | Fertile  Eggs |
| 1 | 160 | 149 |  | 128 | 128 |  | 36 | 31 |  | 69 | 69 |
| 2 | 295 | 289 |  | 0 | 0 |  | 0 | 0 |  | 0 | 0 |
| 3 | 0 | 0 |  | 125 | 125 |  | 247 | 242 |  | 0 | 0 |
| 4 | 0 | 0 |  | 0 | 0 |  | 0 | 0 |  | 0 | 0 |
| 5 | 80 | 80 |  | 0 | 0 |  | 275 | 275 |  | 169 | 169 |
| 6 | 380 | 374 |  | 90 | 87 |  | 0 | 0 |  | 161 | 152 |
| 7 | 0 | 0 |  | 141 | 130 |  | 0 | 0 |  | 93 | 88 |
| 8 | 0 | 0 |  | 307 | 306 |  | 209 | 206 |  | 0 | 0 |
| 9 | 89 | 89 |  | 118 | 113 |  | 312 | 308 |  | 0 | 0 |
| 10 | 397 | 393 |  | 292 | 291 |  | 0 | 0 |  | 0 | 0 |
| 11 | 0 | 0 |  | 354 | 349 |  | 70 | 36 |  | 155 | 154 |
| 12 | 119 | 119 |  | 157 | 155 |  | 62 | 62 |  | 0 | 0 |
| 13 | 262 | 242 |  | 0 | 0 |  | 292 | 288 |  | 545 | 531 |
| 14 | 546 | 544 |  | 0 | 0 |  | 293 | 292 |  | 0 | 0 |
| 15 | 0 | 0 |  | 0 | 0 |  | 0 | 0 |  | 0 | 0 |
| 16 | 0 | 0 |  | 192 | 187 |  | 253 | 250 |  | 451 | 442 |
| 17 | 0 | 0 |  | 265 | 250 |  | 37 | 34 |  | 161 | 160 |
| 18 | 0 | 0 |  | 181 | 166 |  | 81 | 70 |  | 249 | 249 |
| 19 | 714 | 689 |  | 0 | 0 |  | 73 | 66 |  | 121 | 121 |
| 20 | 134 | 88 |  | 0 | 0 |  | 0 | 0 |  | 0 | 0 |
| 21 | 0 | 0 |  | 0 | 0 |  | 74 | 57 |  | 0 | 0 |
| ^1^ Egg production is presented as the total number of eggs present on three spawning tiles per replicate. | | | | | | | | | | | |

Table 3 continued Fecundity and Fertility of Fathead Minnows During the Pulse Dose Exposure to Chlorothalonil

| Day | Solvent Control | | | | | | | | | | |
| --- | --- | --- | --- | --- | --- | --- | --- | --- | --- | --- | --- |
|  | Fecundity and Fertility by Replicate and Day of Exposure | | | | | | | | | | |
|  | Replicate A | |  | Replicate B | |  | Replicate C | |  | Replicate D | |
|  | Eggs  Produced ^1^ | Fertile  Eggs |  | Eggs  Produced ^1^ | Fertile  Eggs |  | Eggs  Produced ^1^ | Fertile  Eggs |  | Eggs  Produced ^1^ | Fertile  Eggs |
| 22 | 0 | 0 |  | 0 | 0 |  | 0 | 0 |  | 0 | 0 |
| 23 | 0 | 0 |  | 287 | 285 |  | 0 | 0 |  | 0 | 0 |
| 24 | 97 | 93 |  | 262 | 259 |  | 0 | 0 |  | 160 | 156 |
| 25 | 212 | 189 |  | 0 | 0 |  | 0 | 0 |  | 70 | 69 |
| 26 | 272 | 258 |  | 301 | 290 |  | 143 | 138 |  | 0 | 0 |
| 27 | 0 | 0 |  | 0 | 0 |  | 380 | 360 |  | 322 | 294 |
| 28 | 0 | 0 |  | 0 | 0 |  | 0 | 0 |  | 192 | 187 |
| 29 | 162 | 152 |  | 123 | 121 |  | 0 | 0 |  | 0 | 0 |
| 30 | 0 | 0 |  | 231 | 224 |  | 0 | 0 |  | 0 | 0 |
| 31 | 0 | 0 |  | 0 | 0 |  | 113 | 111 |  | 0 | 0 |
| 32 | 0 | 0 |  | 0 | 0 |  | 94 | 76 |  | 0 | 0 |
| 33 | 0 | 0 |  | 0 | 0 |  | 11 | 8 |  | 0 | 0 |
| 34 | 633 | 627 |  | 0 | 0 |  | 163 | 162 |  | 0 | 0 |
| 35 | 360 | 338 |  | 493 | 486 |  | 261 | 239 |  | 199 | 198 |
| 36 | 0 | 0 |  | 121 | 116 |  | 188 | 184 |  | 0 | 0 |
| Total: | 4912 | 4713 |  | 4168 | 4068 |  | 3667 | 3495 |  | 3117 | 3039 |
| ^1^ Egg production is presented as the total number of eggs present on three spawning tiles per replicate. | | | | | | | | | | | |

Table 3 continued Fecundity and Fertility of Fathead Minnows During the Pulse Dose Exposure to Chlorothalonil

| Day | Treatment 1 | | | | | | | | | | |
| --- | --- | --- | --- | --- | --- | --- | --- | --- | --- | --- | --- |
|  | Fecundity and Fertility by Replicate and Day of Exposure | | | | | | | | | | |
|  | Replicate A | |  | Replicate B | |  | Replicate C | |  | Replicate D | |
|  | Eggs  Produced ^1^ | Fertile  Eggs |  | Eggs  Produced ^1^ | Fertile  Eggs |  | Eggs  Produced ^1^ | Fertile  Eggs |  | Eggs  Produced ^1^ | Fertile  Eggs |
| 1 | 0 | 0 |  | 168 | 155 |  | 191 | 190 |  | 0 | 0 |
| 2 | 263 | 263 |  | 238 | 197 |  | 214 | 207 |  | 154 | 151 |
| 3 | 23 | 23 |  | 66 | 66 |  | 292 | 289 |  | 0 | 0 |
| 4 | 191 | 189 |  | 193 | 192 |  | 0 | 0 |  | 0 | 0 |
| 5 | 0 | 0 |  | 2 | 2 |  | 127 | 127 |  | 208 | 208 |
| 6 | 308 | 304 |  | 0 | 0 |  | 68 | 65 |  | 249 | 249 |
| 7 | 255 | 249 |  | 596 | 595 |  | 289 | 282 |  | 0 | 0 |
| 8 | 0 | 0 |  | 3 | 3 |  | 0 | 0 |  | 282 | 282 |
| 9 | 93 | 88 |  | 0 | 0 |  | 190 | 190 |  | 261 | 261 |
| 10 | 228 | 226 |  | 0 | 0 |  | 0 | 0 |  | 254 | 254 |
| 11 | 0 | 0 |  | 601 | 593 |  | 147 | 145 |  | 0 | 0 |
| 12 | 321 | 321 |  | 0 | 0 |  | 0 | 0 |  | 112 | 110 |
| 13 | 10 | 10 |  | 0 | 0 |  | 512 | 495 |  | 287 | 287 |
| 14 | 169 | 159 |  | 399 | 399 |  | 119 | 115 |  | 369 | 367 |
| 15 | 185 | 185 |  | 254 | 251 |  | 0 | 0 |  | 261 | 260 |
| 16 | 0 | 0 |  | 125 | 124 |  | 0 | 0 |  | 0 | 0 |
| 17 | 210 | 194 |  | 82 | 81 |  | 0 | 0 |  | 357 | 354 |
| 18 | 155 | 148 |  | 0 | 0 |  | 0 | 0 |  | 244 | 242 |
| 19 | 142 | 113 |  | 0 | 0 |  | 236 | 234 |  | 0 | 0 |
| 20 | 40 | 34 |  | 578 | 548 |  | 378 | 363 |  | 0 | 0 |
| 21 | 412 | 412 |  | 0 | 0 |  | 0 | 0 |  | 218 | 216 |
| ^1^ Egg production is presented as the total number of eggs present on three spawning tiles per replicate. | | | | | | | | | | | |

Table 3 continued Fecundity and Fertility of Fathead Minnows During the Pulse Dose Exposure to Chlorothalonil

| Day | Treatment 1 | | | | | | | | | | |
| --- | --- | --- | --- | --- | --- | --- | --- | --- | --- | --- | --- |
|  | Fecundity and Fertility by Replicate and Day of Exposure | | | | | | | | | | |
|  | Replicate A | |  | Replicate B | |  | Replicate C | |  | Replicate D | |
|  | Eggs  Produced ^1^ | Fertile  Eggs |  | Eggs  Produced ^1^ | Fertile  Eggs |  | Eggs  Produced ^1^ | Fertile  Eggs |  | Eggs  Produced ^1^ | Fertile  Eggs |
| 22 | 0 | 0 |  | 0 | 0 |  | 0 | 0 |  | 212 | 206 |
| 23 | 0 | 0 |  | 0 | 0 |  | 0 | 0 |  | 0 | 0 |
| 24 | 59 | 48 |  | 124 | 124 |  | 0 | 0 |  | 0 | 0 |
| 25 | 64 | 63 |  | 245 | 216 |  | 0 | 0 |  | 0 | 0 |
| 26 | 0 | 0 |  | 210 | 198 |  | 386 | 372 |  | 0 | 0 |
| 27 | 356 | 345 |  | 0 | 0 |  | 0 | 0 |  | 0 | 0 |
| 28 | 186 | 184 |  | 204 | 204 |  | 0 | 0 |  | 349 | 341 |
| 29 | 0 | 0 |  | 353 | 344 |  | 0 | 0 |  | 205 | 200 |
| 30 | 0 | 0 |  | 277 | 272 |  | 0 | 0 |  | 267 | 246 |
| 31 | 498 | 492 |  | 7 | 6 |  | 287 | 287 |  | 205 | 201 |
| 32 | 307 | 306 |  | 216 | 216 |  | 357 | 354 |  | 295 | 294 |
| 33 | 0 | 0 |  | 0 | 0 |  | 0 | 0 |  | 0 | 0 |
| 34 | 0 | 0 |  | 0 | 0 |  | 0 | 0 |  | 0 | 0 |
| 35 | 6 | 6 |  | 0 | 0 |  | 0 | 0 |  | 333 | 294 |
| 36 | 0 | 0 |  | 398 | 393 |  | 0 | 0 |  | 0 | 0 |
| Total: | 4481 | 4362 |  | 5339 | 5179 |  | 3793 | 3715 |  | 5122 | 5023 |
| ^1^ Egg production is presented as the total number of eggs present on three spawning tiles per replicate. | | | | | | | | | | | |

Table 3 continued Fecundity and Fertility of Fathead Minnows During the Pulse Dose Exposure to Chlorothalonil

| Day | Treatment 2 | | | | | | | | | | |
| --- | --- | --- | --- | --- | --- | --- | --- | --- | --- | --- | --- |
|  | Fecundity and Fertility by Replicate and Day of Exposure | | | | | | | | | | |
|  | Replicate A | |  | Replicate B | |  | Replicate C | |  | Replicate D | |
|  | Eggs  Produced ^1^ | Fertile  Eggs |  | Eggs  Produced ^1^ | Fertile  Eggs |  | Eggs  Produced ^1^ | Fertile  Eggs |  | Eggs  Produced ^1^ | Fertile  Eggs |
| 1 | 199 | 185 |  | 0 | 0 |  | 0 | 0 |  | 0 | 0 |
| 2 | 0 | 0 |  | 0 | 0 |  | 286 | 279 |  | 0 | 0 |
| 3 | 0 | 0 |  | 0 | 0 |  | 335 | 328 |  | 106 | 106 |
| 4 | 0 | 0 |  | 409 | 408 |  | 57 | 57 |  | 0 | 0 |
| 5 | 59 | 58 |  | 193 | 193 |  | 124 | 116 |  | 353 | 348 |
| 6 | 101 | 101 |  | 0 | 0 |  | 341 | 334 |  | 239 | 237 |
| 7 | 125 | 123 |  | 0 | 0 |  | 238 | 237 |  | 0 | 0 |
| 8 | 15 | 15 |  | 382 | 380 |  | 0 | 0 |  | 231 | 225 |
| 9 | 11 | 8 |  | 304 | 302 |  | 0 | 0 |  | 0 | 0 |
| 10 | 238 | 234 |  | 217 | 213 |  | 348 | 342 |  | 183 | 178 |
| 11 | 0 | 0 |  | 1 | 1 |  | 62 | 62 |  | 126 | 122 |
| 12 | 0 | 0 |  | 0 | 0 |  | 191 | 191 |  | 174 | 152 |
| 13 | 374 | 368 |  | 0 | 0 |  | 0 | 0 |  | 0 | 0 |
| 14 | 122 | 118 |  | 424 | 415 |  | 0 | 0 |  | 117 | 93 |
| 15 | 0 | 0 |  | 0 | 0 |  | 377 | 375 |  | 164 | 159 |
| 16 | 0 | 0 |  | 299 | 290 |  | 86 | 70 |  | 166 | 145 |
| 17 | 0 | 0 |  | 342 | 340 |  | 0 | 0 |  | 0 | 0 |
| 18 | 139 | 127 |  | 37 | 37 |  | 101 | 84 |  | 0 | 0 |
| 19 | 147 | 132 |  | 168 | 149 |  | 278 | 275 |  | 0 | 0 |
| 20 | 47 | 32 |  | 0 | 0 |  | 141 | 137 |  | 115 | 100 |
| 21 | 2 | 1 |  | 0 | 0 |  | 13 | 11 |  | 166 | 162 |
| Totals: | 1579 | 1502 |  | 2776 | 2728 |  | 2978 | 2898 |  | 2140 | 2027 |
| ^1^ Egg production is presented as the total number of eggs present on three spawning tiles per replicate. | | | | | | | | | | | |

Table 3 continued Fecundity and Fertility of Fathead Minnows During the Pulse Dose Exposure to Chlorothalonil

| Day | Treatment 3 | | | | | | | | | | |
| --- | --- | --- | --- | --- | --- | --- | --- | --- | --- | --- | --- |
|  | Fecundity and Fertility by Replicate and Day of Exposure | | | | | | | | | | |
|  | Replicate A | |  | Replicate B | |  | Replicate C | |  | Replicate D | |
|  | Eggs  Produced ^1^ | Fertile  Eggs |  | Eggs  Produced ^1^ | Fertile  Eggs |  | Eggs  Produced ^1^ | Fertile  Eggs |  | Eggs  Produced ^1^ | Fertile  Eggs |
| 1 | 0 | 0 |  | 110 | 97 |  | 285 | 230 |  | 136 | 130 |
| 2 | 0 | 0 |  | 135 | 129 |  | 0 | 0 |  | 226 | 215 |
| 3 | 352 | 349 |  | 0 | 0 |  | 0 | 0 |  | 0 | 0 |
| 4 | 388 | 388 |  | 356 | 342 |  | 0 | 0 |  | 0 | 0 |
| 5 | 0 | 0 |  | 0 | 0 |  | 0 | 0 |  | 240 | 240 |
| 6 | 0 | 0 |  | 153 | 153 |  | 292 | 292 |  | 176 | 176 |
| 7 | 394 | 389 |  | 3 | 2 |  | 169 | 164 |  | 355 | 345 |
| 8 | 164 | 164 |  | 32 | 28 |  | 256 | 255 |  | 0 | 0 |
| 9 | 0 | 0 |  | 41 | 31 |  | 0 | 0 |  | 0 | 0 |
| 10 | 0 | 0 |  | 85 | 74 |  | 167 | 164 |  | 35 | 35 |
| 11 | 0 | 0 |  | 180 | 173 |  | 146 | 143 |  | 145 | 141 |
| 12 | 281 | 274 |  | 29 | 27 |  | 106 | 104 |  | 0 | 0 |
| 13 | 82 | 80 |  | 90 | 86 |  | 0 | 0 |  | 0 | 0 |
| 14 | 137 | 124 |  | 0 | 0 |  | 0 | 0 |  | 348 | 335 |
| 15 | 146 | 140 |  | 25 | 21 |  | 207 | 201 |  | 130 | 130 |
| 16 | 149 | 140 |  | 182 | 179 |  | 24 | 23 |  | 294 | 288 |
| 17 | 0 | 0 |  | 0 | 0 |  | 0 | 0 |  | 0 | 0 |
| 18 | 0 | 0 |  | 0 | 0 |  | 0 | 0 |  | 0 | 0 |
| 19 | 158 | 144 |  | 30 | 23 |  | 0 | 0 |  | 374 | 374 |
| 20 | 417 | 398 |  | 234 | 210 |  | 174 | 173 |  | 124 | 113 |
| 21 | 15 | 12 |  | 0 | 0 |  | 340 | 337 |  | 0 | 0 |
| Totals: | 2683 | 2602 |  | 1685 | 1575 |  | 2166 | 2086 |  | 2583 | 2522 |
| ^1^ Egg production is presented as the total number of eggs present on three spawning tiles per replicate. | | | | | | | | | | | |

Table 3 continued Fecundity and Fertility of Fathead Minnows During the Pulse Dose Exposure to Chlorothalonil

| Day | Treatment 4 | | | | | | | | | | |
| --- | --- | --- | --- | --- | --- | --- | --- | --- | --- | --- | --- |
|  | Fecundity and Fertility by Replicate and Day of Exposure | | | | | | | | | | |
|  | Replicate A | |  | Replicate B | |  | Replicate C | |  | Replicate D | |
|  | Eggs  Produced ^1^ | Fertile  Eggs |  | Eggs  Produced ^1^ | Fertile  Eggs |  | Eggs  Produced ^1^ | Fertile  Eggs |  | Eggs  Produced ^1^ | Fertile  Eggs |
| 1 | 298 | 283 |  | 296 | 255 |  | 207 | 203 |  | 0 | 0 |
| 2 | 0 | 0 |  | 291 | 253 |  | 122 | 118 |  | 0 | 0 |
| 3 | 0 | 0 |  | 167 | 166 |  | 0 | 0 |  | 115 | 111 |
| 4 | 184 | 174 |  | 0 | 0 |  | 0 | 0 |  | 264 | 230 |
| 5 | 432 | 416 |  | 156 | 142 |  | 0 | 0 |  | 0 | 0 |
| 6 | 0 | 0 |  | 272 | 256 |  | 0 | 0 |  | 0 | 0 |
| 7 | 0 | 0 |  | 0 | 0 |  | 0 | 0 |  | 115 | 112 |
| 8 | 459 | 456 |  | 337 | 336 |  | 0 | 0 |  | 0 | 0 |
| 9 | 0 | 0 |  | 263 | 254 |  | 250 | 250 |  | 499 | 486 |
| 10 | 551 | 550 |  | 245 | 244 |  | 0 | 0 |  | 0 | 0 |
| 11 | 307 | 307 |  | 249 | 229 |  | 370 | 368 |  | 77 | 77 |
| 12 | 206 | 206 |  | 0 | 0 |  | 0 | 0 |  | 0 | 0 |
| 13 | 3 | 3 |  | 0 | 0 |  | 197 | 165 |  | 166 | 163 |
| 14 | 0 | 0 |  | 480 | 463 |  | 184 | 182 |  | 411 | 408 |
| 15 | 235 | 220 |  | 82 | 81 |  | 2 | 2 |  | 0 | 0 |
| 16 | 280 | 274 |  | 177 | 149 |  | 196 | 194 |  | 0 | 0 |
| 17 | 132 | 127 |  | 161 | 154 |  | 0 | 0 |  | 0 | 0 |
| 18 | 187 | 186 |  | 83 | 79 |  | 163 | 158 |  | 70 | 67 |
| 19 | 273 | 269 |  | 466 | 455 |  | 16 | 15 |  | 0 | 0 |
| 20 | 0 | 0 |  | 0 | 0 |  | 0 | 0 |  | 225 | 220 |
| 21 | 184 | 179 |  | 0 | 0 |  | 261 | 261 |  | 0 | 0 |
| Totals: | 3731 | 3650 |  | 3725 | 3516 |  | 1968 | 1916 |  | 1942 | 1874 |
| ^1^ Egg production is presented as the total number of eggs present on three spawning tiles per replicate. | | | | | | | | | | | |

Table 3 continued Fecundity and Fertility of Fathead Minnows During the Pulse Dose Exposure to Chlorothalonil

| Day | Treatment 5 | | | | | | | | | | |
| --- | --- | --- | --- | --- | --- | --- | --- | --- | --- | --- | --- |
|  | Fecundity and Fertility by Replicate and Day of Exposure | | | | | | | | | | |
|  | Replicate A | |  | Replicate B | |  | Replicate C | |  | Replicate D | |
|  | Eggs  Produced ^1^ | Fertile  Eggs |  | Eggs  Produced ^1^ | Fertile  Eggs |  | Eggs  Produced ^1^ | Fertile  Eggs |  | Eggs  Produced ^1^ | Fertile  Eggs |
| 1 | 0 | 0 |  | 215 | 214 |  | 0 | 0 |  | 141 | 132 |
| 2 | 428 | 423 |  | 0 | 0 |  | 0 | 0 |  | 173 | 169 |
| 3 | 334 | 333 |  | 0 | 0 |  | 227 | 221 |  | 0 | 0 |
| 4 | 0 | 0 |  | 0 | 0 |  | 50 | 48 |  | 0 | 0 |
| 5 | 0 | 0 |  | 322 | 322 |  | 241 | 236 |  | 276 | 265 |
| 6 | 169 | 154 |  | 271 | 270 |  | 0 | 0 |  | 229 | 216 |
| 7 | 161 | 146 |  | 191 | 177 |  | 405 | 404 |  | 58 | 51 |
| 8 | 405 | 372 |  | 0 | 0 |  | 62 | 53 |  | 145 | 140 |
| 9 | 96 | 93 |  | 222 | 222 |  | 0 | 0 |  | 153 | 151 |
| 10 | 141 | 136 |  | 183 | 170 |  | 241 | 236 |  | 0 | 0 |
| 11 | 230 | 218 |  | 0 | 0 |  | 50 | 47 |  | 140 | 131 |
| 12 | 16 | 15 |  | 193 | 176 |  | 316 | 302 |  | 0 | 0 |
| 13 | 104 | 87 |  | 296 | 293 |  | 150 | 150 |  | 292 | 286 |
| 14 | 0 | 0 |  | 59 | 57 |  | 0 | 0 |  | 102 | 99 |
| 15 | 0 | 0 |  | 0 | 0 |  | 0 | 0 |  | 158 | 154 |
| 16 | 136 | 133 |  | 0 | 0 |  | 0 | 0 |  | 0 | 0 |
| 17 | 98 | 93 |  | 0 | 0 |  | 190 | 177 |  | 207 | 185 |
| 18 | 87 | 80 |  | 0 | 0 |  | 39 | 39 |  | 0 | 0 |
| 19 | 454 | 433 |  | 8 | 8 |  | 167 | 160 |  | 0 | 0 |
| 20 | 0 | 0 |  | 300 | 288 |  | 0 | 0 |  | 0 | 0 |
| 21 | 0 | 0 |  | 585 | 572 |  | 9 | 9 |  | 99 | 93 |
| 22 | 0 | 0 |  | 0 | 0 |  | 0 | 0 |  | 79 | 79 |
| Totals: | 2859 | 2716 |  | 2845 | 2769 |  | 2147 | 2082 |  | 2252 | 2151 |
| ^1^ Egg production is presented as the total number of eggs present on three spawning tiles per replicate. | | | | | | | | | | | |

Table 4 Replicate Totals for Reproduction of Fathead Minnows From a Pulse Dose Exposure to Chlorothalonil

| Treatment | Rep. | Cumulative  Number of Eggs Produced ^1^ | Number of  Fertile Eggs | Percent  Fertility ^2^ | Number of Reproductive  Days ^3^ | Eggs / Female Reproductive  Day ^2^ |
| --- | --- | --- | --- | --- | --- | --- |
| Negative Control | A | 5336 | 5186 | 97.2 | 144 | 37.1 |
|  | B | 4216 | 4109 | 97.5 | 135 | 31.2 |
|  | C | 4748 | 4671 | 98.4 | 144 | 33.0 |
|  | D | 3954 | 3834 | 97.0 | 144 | 27.5 |
|  |  |  |  |  |  |  |
| Solvent Control | A | 4912 | 4713 | 95.9 | 144 | 34.1 |
|  | B | 4168 | 4068 | 97.6 | 144 | 28.9 |
|  | C | 3667 | 3495 | 95.3 | 144 | 25.5 |
|  | D | 3117 | 3039 | 97.5 | 144 | 21.6 |
|  |  |  |  |  |  |  |
| Treatment 1 | A | 4481 | 4362 | 97.3 | 144 | 31.1 |
|  | B | 5339 | 5179 | 97.0 | 144 | 37.1 |
|  | C | 3793 | 3715 | 97.9 | 144 | 26.3 |
|  | D | 5122 | 5023 | 98.1 | 144 | 35.6 |
|  |  |  |  |  |  |  |
| Treatment 2 | A | 1579 | 1502 | 95.1 | 68 | 23.5 |
|  | B | 2776 | 2728 | 98.3 | 84 | 33.0 |
|  | C | 2978 | 2898 | 97.3 | 84 | 35.5 |
|  | D | 2140 | 2027 | 94.7 | 84 | 25.5 |
| ^1^ Total number of eggs collected on three spawning tiles per replicate.  ^2^ Calculated using Excel; manual calculations may differ slightly.  ^3^ The maximum number of reproductive days per replicate in negative control, solvent control and Treatment 1 is 144 (36 days x 4 females per replicate) and Treatment 2 is 84 (21 days x 4 females per replicate). | | | | | | |

Table 4 continued Replicate Totals for Reproduction of Fathead Minnows From a Pulse Dose Exposure to Chlorothalonil (continued)

| Treatments | Rep. | Cumulative  Number of Eggs Produced ^1^ | Number of  Fertile Eggs | Percent  Fertility ^2^ | Number of Reproductive  Days ^3^ | Eggs / Female Reproductive  Day ^2^ |
| --- | --- | --- | --- | --- | --- | --- |
| Treatment 3 | A | 2683 | 2602 | 97.0 | 84 | 31.9 |
|  | B | 1685 | 1575 | 93.5 | 84 | 20.1 |
|  | C | 2166 | 2086 | 96.3 | 80 | 27.1 |
|  | D | 2583 | 2522 | 97.6 | 84 | 30.7 |
|  |  |  |  |  |  |  |
| Treatment 4 | A | 3731 | 3650 | 97.8 | 84 | 44.4 |
|  | B | 3725 | 3516 | 94.4 | 84 | 44.3 |
|  | C | 1968 | 1916 | 97.4 | 84 | 23.4 |
|  | D | 1942 | 1874 | 96.5 | 70 | 27.7 |
|  |  |  |  |  |  |  |
| Treatment 5 | A | 2859 | 2716 | 95.0 | 88 | 32.5 |
|  | B | 2845 | 2769 | 97.3 | 88 | 32.3 |
|  | C | 2147 | 2082 | 97.0 | 88 | 24.4 |
|  | D | 2252 | 2151 | 95.8 | 88 | 25.6 |
|  |  |  |  |  |  |  |
| ^1^ Total number of eggs collected on three spawning tiles per replicate.  ^2^ Calculated using Excel; manual calculations may differ slightly.  ^3^ The maximum number of reproductive days per replicate in Treatment 3 and 4 is 84 (21 days x 4 females per replicate) and in Treatment 5 is 88 (22 days x 4 females per replicate). | | | | | | |
